# Inspection and polypectomy during both insertion and withdrawal or only during withdrawal of colonoscopy? A protocol for systematic review and meta analysis

**DOI:** 10.1097/MD.0000000000020775

**Published:** 2020-07-02

**Authors:** Yaping Wei, Guofan Shen, Yutong Yang, Zheng Jin, Wei Hu, Ying Zhu

**Affiliations:** Affiliated Hangzhou First People's Hospital, Zhejiang University School of Medicine, Hangzhou, China.

**Keywords:** adenoma detection rate, colonoscopy, insertion phase, polypectomy

## Abstract

**Introduction:**

Current evidence supporting additional inspection and polypectomy during insertion of colonoscopy is limited. We plan to provide a systematic review and meta-analysis to compare the yield of inspection and polypectomy during both insertion and withdrawal versus the traditional practice of inspection and polypectomy during withdrawal only.

**Methods and analysis:**

Randomised controlled trials evaluating inspection and polypectomy during both insertion and withdrawal versus inspection and polypectomy during withdrawal only will be searched in MEDLINE, EMBASE, Web of Science, the Cochrane Library, ClinicalTrials.gov, and Google Scholar, from database inception to 31 May 2020. Data on study design, participant characteristics, and outcomes will be extracted. Primary outcomes to be assessed are adenoma detection rate. Secondary outcomes include polyp detection rate, advanced adenoma detection rate, the mean number of adenomas per patient, polyp miss rate, the mean number of adenomas per colonoscopy, procedure duration, cecal intubation rate, procedure difficulty, patient discomfort, sedation dose, and adverse events. Study quality will be assessed using the Cochrane Risk of Bias Tool. Meta-analysis will be performed using RevMan V.5.3 statistical software. Data will be combined with random effect model. The results will be presented as a risk ratio (RR) for dichotomous data, and weighted/standard mean difference for continuous data. Publication bias will be visualized using funnel plots.

**Ethics and dissemination:**

This study will not use primary data, and therefore formal ethical approval is not required. The findings will be disseminated through peer-reviewed journals and committee conferences.

**Protocol registration number:**

INPLASY202050051.

## Abstract 2

Supplemental Digital Content is available in the text

## 1 Introduction

Colonoscopy with diagnosis and treatment of polyps is considered to be a powerful tool to reduce the incidence and mortality rate of colorectal cancer (CRC).^[1]^ Colonoscopy is typically performed with rapid advance of the endoscope to the cecum and then performing a thorough inspection and polypectomy during withdrawal. Polyps detected during insertion are commonly removed during withdrawal. However, many colonoscopists have recognized that polyps seen but not removed during insertion are sometimes difficult to find during withdrawal.^[1]^ This may cause adenoma missed and contribute to the development of interval CRC.^[3,4]^ Several studies have investigated whether additional inspection and polypectomy during insertion affect colonoscopy quality and time efficiency.^[3,4]^ The conclusions of these studies were controversial. We had planned to conduct a systematic review and meta-analysis to compare the yield of inspection and polypectomy during both insertion and withdrawal (IW) versus the traditional practice of inspection and polypectomy during withdrawal only (WO).

## 2 Methods

The review will be performed according to the recommendations specified in the Cochrane Handbook for Intervention Reviews.^[11]^ The reporting of the review will follow the Preferred Reporting Items for Systematic Reviews and Meta-Analysis statement.^[12]^

### 2.1 Criteria for considering studies for this review

Eligibility criteria are established in terms of the Population-Intervention-Comparison-Outcome-Study design framework. Studies will be selected according to the following criteria:

#### 2.1.1 Participants

Included studies will involve patients undergoing colonoscopy, with no age limitation. There will be no restrictions on colonoscopy indications, which include screening, surveillance, and diagnostic colonoscopy. Patients with previous colonic resection, and those with suboptimal bowel preparation will be excluded.

#### 2.1.2 Interventions/comparison

The intervention comparisons are IW vs WO. In IW group, the endoscopist will remove visible polyps instantly regardless of whether the colonoscope is being advanced to the cecum or pulled back. In WO group, the endoscopist will advance the endoscope to the cecum without removing polyps. Careful inspection for polyps and polypectomy will be performed entirely during withdrawal. In both groups, the endoscopists will be instructed to focus on rapid insertion.

#### 2.1.3 Outcomes

Primary outcome: adenoma detection rate (ADR) (defined as the percentage of colonoscopies with at least one adenoma).^[13]^

Secondary outcomes: polyp detection rate (defined as the percentage of colonoscopies with at least one polyp), advanced ADR (defined as proportion of colonoscopies where at least one advanced adenoma was found), the mean number of adenomas per patient, polyp miss rate (defined as the ratio between the number of polyps detected during insertion but missed during withdrawal over the total amount of polyps detected during both insertion and withdrawal), the mean number of adenomas per colonoscopy,^[14]^ cecal intubation rate, colonoscopist’ perception of difficulty of procedure, procedure times (insertion, withdrawal, and total), patients’ assessment of discomfort during colonoscopy, sedation dose, and adverse events.

#### 2.1.4 Study design

Only randomized clinical trials (RCTs) will be included. Unpublished trials and abstracts will not be included. We will only include studies that are presented in English language due to constraints in translational resources.

Exclusion criteria will be:

(1) non-controlled studies, observational studies, case reports, reviews, editorials and letters to editor;
(2) duplicate studies, or animal studies;
(3) no data on any of the primary or secondary outcomes.

### 2.2 Search methods for identification of studies

#### 2.2.1 Electronic searches

Two investigators (ZJ and YW) will independently search MEDLINE, EMBASE, Web of Science, the Cochrane Library, ClinicalTrials.gov, and Google Scholar, for all entries through 31 May 2020. The search strategies will be decided on after a discussion among all reviewers. The primary search strategy will be used for PubMed MEDLINE (Online Supplementary Appendix I). Modifications to the search strategy will be made for other databases. We will assess eligibility of the retrieved articles by title and abstract using predetermined inclusion criteria. If this information is insufficient for eligibility assessment, we will review the full article. If any up-to-date evidence is published during the review period, we will evaluate the eligibility of each study and consider its addition to the analysis.

#### 2.2.2 Searching other resources

To further increase the robustness of the literature search, a manual recursive search of the reference sections of the retrieved articles, as well as the related articles option in PubMed, will be carried out to identify other potentially relevant articles.

### 2.3 Data collection and analysis

#### 2.3.1 Selection of studies

**Figure 1:**
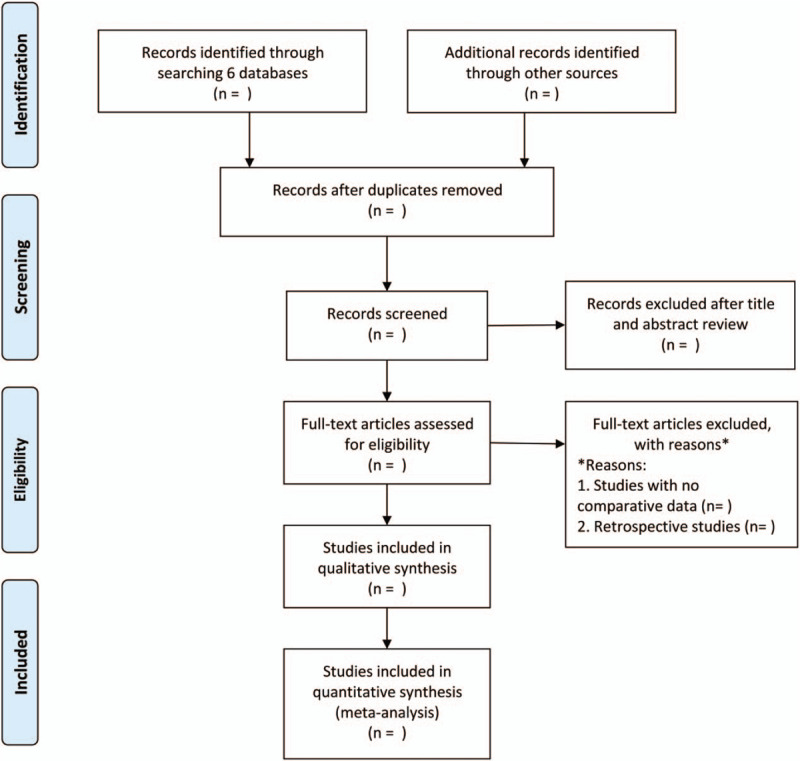
Flow diagram of the study selection process.

Decisions about study inclusion and exclusion will be made independently by 2 investigators (YW and YY). Disagreements will be resolved by consensus after a mutual discussion. The details of the study selection procedure are shown in a the Preferred Reporting Items for Systematic Reviews and Meta-Analysis flow chart. (Fig. 1)

#### 2.3.2 Data extraction and management

Two investigators (YW and GS) will independently extract the appropriate data onto a data collection form (Online Supplementary Appendix II). The following variables will be contained in the collection form: author, year of publication, country of origin, study design, number of centers, number of participating endoscopists, patient demographics, indications for colonoscopy, and study outcomes. When necessary data are not included in the published studies, the corresponding authors will be contacted for additional information. If there is no reply, we will analyze only the available data. If there is no data on any of the primary or secondary outcomes, those studies will be excluded from the meta-analyses.

#### 2.3.3 Assessment of risk of bias in included studies

We will assign two independent investigators (YW and ZJ) to appraise methodological quality of the included trials with the Cochrane Collaboration’ s tool for assessing risk of bias.^[15]^ The tool appraises existence of selection bias by assessing methods of randomization and allocation concealment, performance and detection of biases by checking blinding of personnel and outcome assessment, and attrition and reporting bias by evaluating incomplete and selective data reporting. Each of the item is assigned a judgment of high, low, or unclear risk.

#### 2.3.4 Data synthesis

Standard mean differences will be calculated for continuous variables including patient discomfort and procedure difficulty based on different visual analog scales. Weighted mean differences will be calculated for continuous variables. Medians will be used if means are not available and standard deviations will be calculated or imputed when possible.^[16,17]^ Risk ratios will be calculated for categorical variables. Owning to the assumption of inherently various study scenarios and study populations, a random effects model for all analyses will be assumed. Heterogeneity among studies will be assessed by calculating the *I*^2^ statistics whereby *I*^2^ < 25% indicates no heterogeneity, 25%≤ *I*^2^ < 50% indicates mild heterogeneity, 50%≤ *I*^2^ < 75% indicates moderate heterogeneity and *I*^2^ ≥75% indicates strong heterogeneity.^[18]^ We had planned that if sufficient studies (≥10) are included in the analysis of primary outcomes, we would construct funnel plots to evaluate publication bias,^[11]^ otherwise, Egger test will be applied.^[19]^ All statistical analyses will be performed using Review Manager 5.3 (The Cochrane Collaboration, The Nordic Cochrane Centre, Copenhagen, Denmark).

#### 2.3.5 Subgroup analyses

In the case of possible strong heterogeneity, we will explore the possible sources using subgroup analyses. Subgroup analyses will be carried out based on study setting, study origin, insufflated gas during colonoscopy, the level of ADR with conventional examination method (WO group), and colonoscopy indication. For those subgroups with only 1 study included, subgroup analyses will not be performed.

#### 2.3.6 Sensitivity analysis

We will carry out a sensitivity analysis by systematically removing every study and checking the pooled results for the remaining studies to see if there is any significant change in test performance.

#### 2.3.7 Confidence in cumulative evidence

The quality of evidence will be assessed using the Grading of Recommendations Assessment, Development and Evaluation (GRADE) approach.^[20]^ This will be done independently by two reviewers (YW, GS). If there is a discrepancy, it will be resolved by discussion or a third reviewer as needed. The quality of evidence will be graded as high, moderate, low or very low, and the GRADE pro platform will be used to summarise the findings.

### 2.4 Patient and public involvement

Because the collected data within this systematic review and meta-analysis originates from previously published studies, patients and the general public were not involved in the development of the research question or choice of outcome measures that we wanted to assess.

## 3 Discussion

Colonoscopy is considered to be the preferred modality for CRC screening.^[21]^ Polyps detected during insertion of colonoscopy are commonly removed during withdrawal. However, these polyps, especially small polyps, are not easily found during withdrawal.^[2,22]^ Several RCTs have investigated the yield of IW versus WO showing inconsistent results.^[5–10]^ We therefore propose a meta-analysis to pool the evidence to evaluate the performance of the 2 examination strategies.

One strength of our meta-analysis will be that we will assess multiple clinically relevant outcomes in the comparison of IW and WO. Comprehensive subgroup analyses will be performed to identify knowledge gaps that require further research. This will be the first meta-analysis of RCTs assessing the yield of additional inspection and polypectomy during insertion of colonoscopy. The results of this study will influence clinical practice for colonoscopy, assist in future guideline development and guide future research endeavors.

## Author contributions

YZ is the guarantor. YW drafted the manuscript protocol. YW, GS, and YY contributed to the development of the selection criteria, article screening strategy, risk of bias assessment strategy and data extraction criteria. ZJ developed the search strategy. WH provided statistical expertise. All authors read, provided feedback and approved the final protocol.

**Supervision:** Ying Zhu.

## Supplementary Material

Supplemental Digital Content
