# Supplementary material for: Inspection and polypectomy during both insertion and withdrawal or only during withdrawal of colonoscopy? A protocol for systematic review and meta analysis
Source: Medicine (Baltimore). 2020 Jul 2;99(27):e20775. doi: 10.1097/MD.0000000000020775 (PMC7337486; doi:10.1097/MD.0000000000020775)
Supplement: Supplemental Digital Content [file medi-99-e20775-s001.docx]

Appendix I. **PubMed-MEDLINE search strategy**

up to 31 May 2020.

#1 "Colonoscopy"[MeSH] OR "colonoscopes"[tiab] OR "polypectomy"[tiab]

#2 "insertion*"[tiab] and "withdraw* "[tiab]

#3 #1 AND #2

#4 "randomized controlled trial"[pt] OR "controlled clinical trial"[pt] OR "randomized"[tiab] OR "randomised"[tiab] OR "randomly"[tiab]

#5 #3 AND #4

Appendix II. **Data extraction form**

| Article title |  | |
| --- | --- | --- |
| First author |  | |
| Year of publication |  | |
| Location |  | |
| Setting |  | |
| Study design |  | |
| Colonoscopists, n |  | |
| Experience of colonoscopists |  | |
| Sedation regimens |  | |
| Insufflated gas |  | |
| Study quality |  | |
| Patient characteristics | IW | WO |
| Sample size, n |  |  |
| Age (y), mean ± SD |  |  |
| Sex, male, % |  |  |
| Colonoscopy indications (n) |  |  |
| **Adenoma detection rate, n/N (%)** |  |  |
| **Polyp detection rate, n/N (%)** |  |  |
| **Advanced adenoma detection rate, n/N (%)** |  |  |
| **Mean number of adenomas per patient, n/N (%)** |  |  |
| **Mean number of adenomas per colonoscopy, n/N (%)** |  |  |
| **Polyp miss rate, n/N (%)** |  |  |
| **Procedure time (min), mean ± SD** |  |  |
| **Total procedure time** |  |  |
| **Insertion time** |  |  |
| **Withdrawal time** |  |  |
| **Cecal intubation rate, n/N (%)** |  |  |
| **Procedure difficulty, mean ± SD** |  |  |
| **Patient discomfort, mean ± SD** |  |  |
| **Sedation doses, mean ± SD** |  |  |
| **Adverse events, n/N (%)** |  |  |
| IW, inspection and polypectomy during both insertion and withdrawal; SD, standard deviation; WO, inspection and polypectomy during withdrawal only.  Bold items are outcomes of our analysis. | | |
